# Supplementary material for: CSF total tau as a proxy of synaptic degeneration
Source: Nat Commun. 2025 Aug 29;16:8076. doi: 10.1038/s41467-025-63545-5 (PMC12397218; doi:10.1038/s41467-025-63545-5)
Supplement: Supplementary file 1 — Supplementary Information [file 41467_2025_63545_MOESM1_ESM.pdf]

Supplementary Material

Index

Supplementary material	Content
**Table 1**	Demographics by cohort – Main analysis
**Table 2**	ADNI Demographics at tau PET visit
**Figure 1**	Correlation matrices by cohort
**Table 3**	Correlation estimates CU/CI - overall
**Table 4**	Correlation estimates – ADNI cohort
**Table 5**	Correlation estimates – WRAP cohort
**Table 6**	Beta-estimates CU - overall
**Table 7**	Beta-estimates with FDG-PET CU - overall
**Figure 2**	Scatter plots of main associations with CSF t-tau
**Figure 3**	Beta-estimates CU/CI – overall by sex and clinical diagnosis
**Table 8**	Beta-estimates CU – overall by sex
**Figure 4**	Beta-estimates CU/CI – CSF p-tau181 and plasma p-tau217
**Figure 5**	Beta-estimates by cohort
**Table 9**	Beta-estimates CU/CI – CSF p-tau181 and plasma p-tau217
**Table 10**	Beta-estimates CU/CI – ADNI cohort
**Table 11**	Beta-estimates CU – WRAP cohort
**Table 12**	Beta-estimates CI - overall
**Table 13**	Beta-estimates with FDG-PET CI - overall
**Table 14**	Beta-estimates CI – overall by sex
**Table 15**	Beta-estimates – overall by clinical diagnosis
**Table 16**	pR2 CU/CI – ADNI cohort
**Table 17**	pR2 CU– WRAP cohort
**Figure 6**	Group (HCV/SNAP25), NfL/Ng, NfL/SNAP25 – all cohorts
**Table 18**	Group (HCV/Ng) – all cohorts
**Table 19**	Group (HCV/SNAP25) – all cohorts
**Table 20**	Group (NfL/Ng) – all cohorts
**Table 21**	Group (NfL/SNAP25) – all cohorts
**Table 22**	Group (HCV/Ng) – ADNI cohort
**Table 23**	Group (HCV/SNAP25) – ADNI cohort
**Table 24**	Group (HCV/Ng) – WRAP cohort
**Table 25**	Group (HCV/SNAP25) – WRAP cohort

Supplementary Table 1. Demographics and key characteristics by cohort

Characteristics	WRAP (n = 285)		ADNI (n=1,383)		
	CU (n = 269)	CI (n = 16)	CU (n = 483)	CI (n = 900)	P-value
Age, years	65 (± 7)	72 (± 5.3)	72 (± 6.3)	73 (± 7.8)	0.233
Females	186 (69 %)	11 (69 %)	276 (57 %)	366 (41 %)	< 0.001
Race					
White	253 (94 %)	15 (94 %)	439 (91 %)	853 (95 %)	0.006
Unknown	4 (1.5 %)	0 (0 %)	0 (0 %)	3 (0.3 %)	0.556
Ethnicity					
Not Hispanic/Latino	265 (99 %)	16 (100 %)	462 (96 %)	867 (96 %)	0.561
Unknown	0 (0 %)	0 (0 %)	3 (0.6 %)	4 (0.4 %)	0.700
APOEe4 carriers	100 (37 %)	7 (44 %)	161 (33 %)	508 (56 %)	< 0.001
Education, years	16 (± 2.4)	15 (± 2.4)	17 (± 2.4)	16 (± 2.8)	< 0.001
Meta-temporal ROI tau PET	1.1 (± 0.19)	1.3 (± 0.34)	-	-	-
HCV (cm^3)	4.0 (± 0.36)	3.6 (± 0.42)	3.7 (± 0.39)	3.3 (± 0.56)	< 0.001
CSF $A\beta_{1-42}$ (pg/ml)	1000 (± 420)	960 (± 410)	1200 (± 570)	800 (± 380)	< 0.001
CSF NfL (pg/ml)	100 (± 51)	180 (± 94)	1100 (± 420)	1600 (± 970)	< 0.001
CSF t-tau (pg/ml)	210 (± 73)	260 (± 120)	230 (± 92)	310 (± 140)	< 0.001
CSF Ng (pg/ml)	900 (± 370)	1100 (± 530)	340 (± 230)	530 (± 320)	< 0.001
CSF SNAP25 (pg/ml)	2.3 (± 0.75)	3.2 (± 1.6)	4.5 (± 1.4)	5.7 (± 2.2)	0.001

Values are mean (± s.d.) for continuous variables and n (%) for categorical variables. Continuous variables were tested with two-sided Student's t-test. Categorical variables were tested with Fischer's exact test. Missing values: APOEe4 (WRAP: n = 20, ADNI: n = 38); tau PET (WRAP: n = 176); HCV (ADNI: n = 67); CSF NfL (ADNI: n = 1040); CSF Ng (ADNI: n = 1054); CSF SNAP25 (ADNI: n = 1253). P-values refer to the comparison between CU and CI within cohort. Cognitively unimpaired (CU). Cognitively impaired (CI). Amyloid-beta (Aβ). CSF (cerebrospinal fluid). Total-tau (T-tau). Hippocampal volume (HCV). Neurofilament light chain protein (NfL). Neurogranin (Ng). Synaptosomal-associated protein 25 (SNAP25). Positron emission tomography (PET). Cohorts: Alzheimer's Disease Neuroimaging Initiative (ADNI). Wisconsin Registry for Alzheimer's Prevention (WRAP).

Supplementary Table 2. Demographics and key characteristics of subset of ADNI participants with tau PET

Characteristics	ADNI (n = 470)		
	**CU (n = 265)**	**CI (n = 205)**	**P-value**
Age, years	70 (± 6.3)	72 (± 7.5)	0.042
Females	161 (61 %)	87 (42 %)	< 0.001
Race			
White	243 (92 %)	192 (94 %)	0.481
Unknown	0 (9 %)	1 (0.48 %)	0.436
Ethnicity			
Not Hispanic/Latino	252 (95 %)	200 (98 %)	0.226
Unknown	2 (0.75 %)	0 (0 %)	0.507
APOEe4 carriers	94 (35 %)	91 (44 %)	0.008
Education, years	17 (± 2.2)	16 (± 2.5)	0.001
Meta-temporal ROI tau PET	1.8 (± 0.26)	2.4 (± 0.93)	< 0.001
HCV (cm^3)	2.2 (± 0.29)	1.9 (± 0.35)	< 0.001
CSF $A\beta_{1-42}$ (pg/ml)	1300 (± 640)	900 (± 550)	< 0.001
CSF t-tau (pg/ml)	240 (± 92)	300 (± 130)	< 0.001

Values are mean (± s.d.) for continuous variables and n (%) for categorical variables. Continuous variables were tested with Student's t-test. Categorical variables were tested with Fischer's exact test. Missing values: APOEe4 (n = 20); HCV (n = 99). P-values refer to the comparison between CU and CI. Cognitively unimpaired (CU). Cognitively impaired (CI). Amyloid-beta (Aβ). CSF (cerebrospinal fluid). Total-tau (T-tau). Hippocampal volume (HCV). Neurofilament light chain protein (NfL). Neurogranin (Ng). Synaptosomal-associated protein 25 (SNAP25). Positron emission tomography (PET). Cohorts: Alzheimer's Disease Neuroimaging Initiative (ADNI).

Supplementary Figure 1. CSF t-tau is more closely related to synaptic than neuronal degeneration in two independent cohorts.

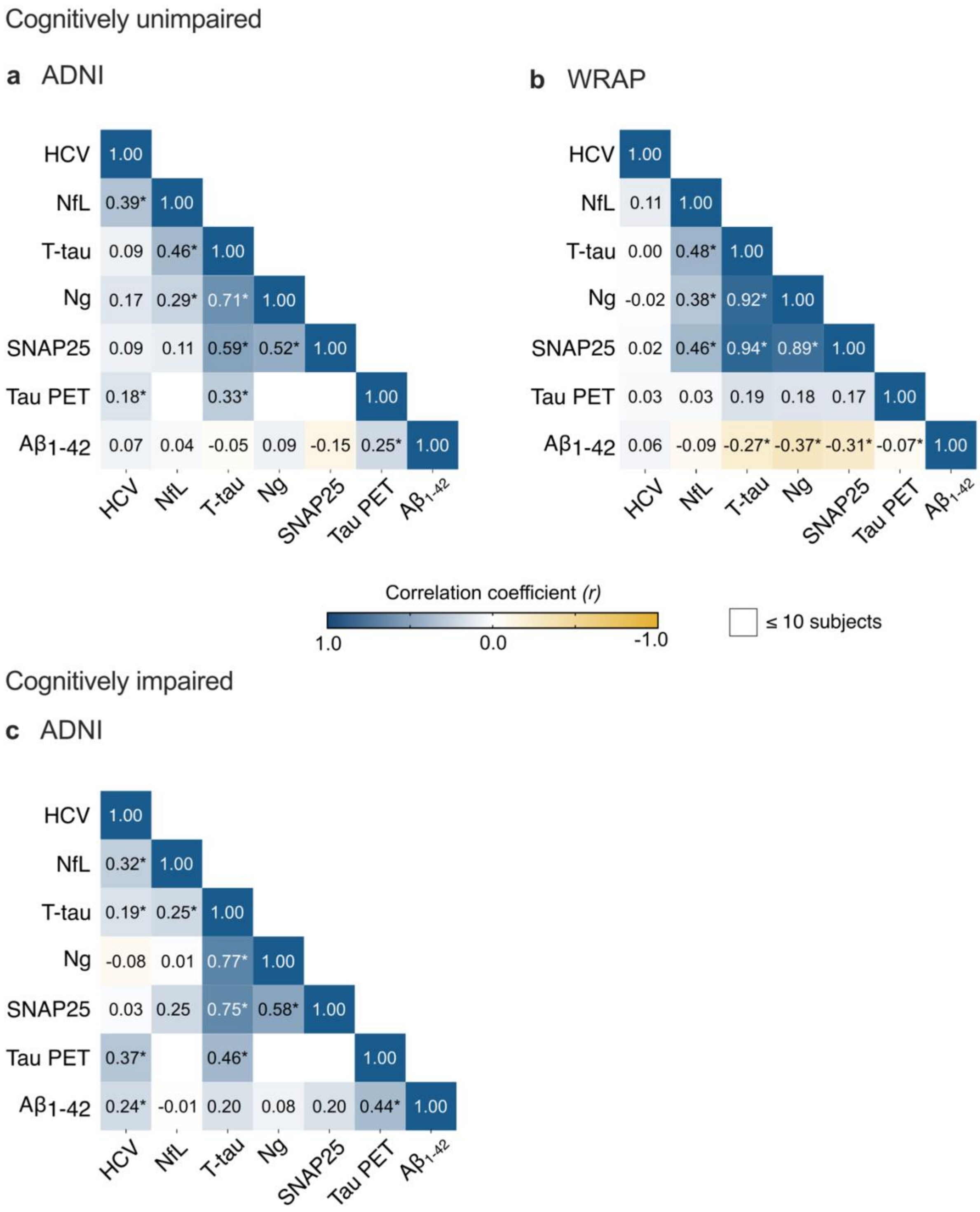

Correlation matrix shows significant two-sided Pearson's coefficient correlations after correcting for multiple comparisons (*padj-value < 0.05) among established biomarkers of neurodegeneration (HCV, CSF NfL and t-tau), synaptic (CSF Ng and SNAP25), and tau (tau PET) and amyloid (CSF $A\beta_{1\text{-}42}$) pathologies in **(a-b)** CU and **(c)** CI individuals in each cohort. Data points with sample sizes of 10 or less are omitted and displayed in white. Cognitively unimpaired (CU). Cognitively impaired (CI). Amyloid-β (Aβ). CSF (cerebrospinal fluid). Total-tau (T-tau). Hippocampal volume (HCV). Neurofilament light chain protein (NfL). Neurogranin (Ng). Synaptosomal-associated protein 25 (SNAP25). Positron emission tomography (PET). Cohorts: Alzheimer's Disease Neuroimaging Initiative (ADNI). Wisconsin Registry for Alzheimer's Prevention (WRAP). Source data are provided as a Source Data file.

Supplementary Table 3. Correlation matrix estimates for synaptic, neurodegeneration and tau pathology biomarkers in cognitively unimpaired and impaired individuals across two independent cohorts.

Biomarkers	Pearson r CU	P values CU	P_{adj} CU	N CU	Pearson r CI	P values CI	P_{adj} CI	N CI
HCV and NfL	0.192 [0.089 to 0.290]	<0.001	0.002	353	0.324 [0.207 to 0.432]	<0.001	<0.001	245
HCV and T-tau	0.056 [-0.018 to 0.128]	0.136	0.949	720	0.189 [0.124 to 0.254]	<0.001	<0.001	847
HCV and Ng	0.016 [-0.089 to 0.121]	0.763	1.000	347	-0.062 [-0.188 to 0.066]	0.343	1.000	237
HCV and SNAP25	0.043 [-0.099 to 0.183]	0.554	1.000	192	0.018 [-0.192 to 0.227]	0.866	1.000	88
HCV and Tau PET	0.140 [0.030 to 0.246]	0.013	0.088	318	0.366 [0.222 to 0.494]	<0.001	<0.001	157
HCV and $A\beta_{1-42}$	0.066 [-0.008 to 0.138]	0.079	0.553	720	0.236 [0.172 to 0.299]	<0.001	<0.001	847
NfL and T-tau	0.472 [0.387 to 0.549]	<0.001	<0.001	355	0.266 [0.152 to 0.373]	<0.001	<0.001	273
NfL and Ng	0.349 [0.254 to 0.438]	<0.001	<0.001	349	0.029 [-0.092 to 0.149]	0.641	1.000	264
NfL and SNAP25	0.382 [0.255 to 0.497]	<0.001	<0.001	193	0.315 [0.117 to 0.488]	0.002	0.016	92
NfL and Tau PET	0.035 [-0.164 to 0.231]	0.732	1.000	99	NA	NA	NA	10
NfL and $A\beta_{1-42}$	-0.049 [-0.152 to 0.055]	0.356	1.000	355	-0.028 [-0.146 to 0.091]	0.651	1.000	273
T-tau and Ng	0.886 [0.861 to 0.906]	<0.001	<0.001	349	0.792 [0.743 to 0.833]	<0.001	<0.001	265
T-tau and SNAP25	0.868 [0.828 to 0.899]	<0.001	<0.001	193	0.776 [0.679 to 0.846]	<0.001	<0.001	93
T-tau and Tau PET	0.296 [0.199 to 0.388]	<0.001	<0.001	357	0.439 [0.323 to 0.541]	<0.001	<0.001	213
T-tau and $A\beta_{1-42}$	-0.136 [-0.205 to -0.065]	<0.001	<0.001	761	0.196 [0.134 to 0.257]	<0.001	<0.001	931
Ng and SNAP25	0.828 [0.777 to 0.868]	<0.001	<0.001	189	0.632 [0.489 to 0.742]	<0.001	<0.001	90
Ng and Tau PET	0.179 [-0.018 to 0.364]	0.076	0.530	99	NA	NA	NA	10
Ng and $A\beta_{1-42}$	-0.241 [-0.338 to -0.140]	<0.001	<0.001	349	0.096 [-0.028 to 0.214]	0.119	0.835	265
SNAP25 and Tau PET	0.175 [-0.033 to 0.369]	0.099	0.695	90	NA	NA	NA	7
SNAP25 and $A\beta_{1-42}$	-0.274 [-0.400 to -0.138]	<0.001	0.001	193	0.120 [-0.086 to 0.316]	0.251	1.000	93
$A\beta_{1-42}$ and Tau PET	0.185 [0.083 to 0.284]	<0.001	0.003	357	0.445 [0.330 to 0.546]	<0.001	<0.001	213

Two-sided Pearson correlation coefficients (r) along with 95% confidence intervals, p-values, p-values adjusted for multiple comparisons (P_{adj}) and sample sizes for the correlation tests between synaptic (CSF Ng and SNAP25), neurodegeneration (HCV, CSF NfL and t-tau), and tau and amyloid pathologies (Tau PET and CSF $A\beta_{1-42}$) biomarkers in cognitively unimpaired (CU) and impaired (CI) individuals across two independent cohorts (ADNI and WRAP). HCV and amyloid-β ($A\beta_{1-42}$) were inverted.

Supplementary Table 4. Correlation matrix estimates for synaptic, neurodegeneration and tau pathology biomarkers in cognitively unimpaired and impaired individuals in the ADNI cohort.

Biomarkers	Pearson r CU	P values CU	P_{adj} CU	N CU	Pearson r CI	P values CI	P_{adj} CI	N CI
HCV and NfL	0.394 [0.207 to 0.561]	<0.001	<0.001	84	0.317 [0.195 to 0.429]	<0.001	<0.001	229
HCV and T-tau	0.086 [-0.00645 to 0.177]	0.068	0.068	451	0.190 [0.123 to 0.254]	<0.001	<0.001	831
HCV and Ng	0.166 [-0.0588 to 0.375]	0.146	0.146	78	-0.077 [-0.207 to 0.0551]	0.252	1.000	221
HCV and SNAP25	0.088 [-0.215 to 0.375]	0.572	0.572	44	0.025 [-0.196 to 0.244]	0.824	1.000	80
HCV and Tau PET	0.177 [0.0459 to 0.303]	0.009	0.009	219	0.374 [0.226 to 0.506]	<0.001	<0.001	147
HCV and $A\beta_{1-42}$	0.074 [-0.0182 to 0.165]	0.115	0.115	451	0.238 [0.172 to 0.301]	<0.001	<0.001	831
NfL and T-tau	0.457 [0.271 to 0.610]	<0.001	<0.001	86	0.255 [0.136 to 0.366]	<0.001	<0.001	257
NfL and Ng	0.286 [0.0709 to 0.476]	0.010	0.050	80	0.007 [-0.117 to 0.132]	0.910	1.000	248
NfL and SNAP25	0.113 [-0.187 to 0.393]	0.462	1.000	45	0.255 [0.0427 to 0.445]	0.019	0.097	84
NfL and Tau PET	NA	NA	NA	NA	NA	NA	NA	NA
NfL and $A\beta_{1-42}$	0.037 [0.271 to 0.610]	0.734	1.000	86	-0.015 [-0.137 to 0.108]	0.812	1.000	257
T-tau and Ng	0.706 [0.576 to 0.802]	<0.001	<0.001	80	0.775 [0.720 to 0.820]	<0.001	<0.001	249
T-tau and SNAP25	0.590 [0.359 to 0.753]	<0.001	<0.001	45	0.750 [0.638 to 0.830]	<0.001	<0.001	85
T-tau and Tau PET	0.330 [0.216 to 0.434]	<0.001	<0.001	258	0.463 [0.348 to 0.565]	<0.001	<0.001	203
T-tau and $A\beta_{1-42}$	-0.053 [-0.141 to 0.0354]	0.239	1.000	492	0.198 [0.135 to 0.259]	<0.001	<0.001	915
Ng and SNAP25	0.525 [0.259 to 0.717]	<0.001	0.002	41	0.580 [0.415 to 0.708]	<0.001	<0.001	82
Ng and Tau PET	NA	NA	NA	NA	NA	NA	NA	NA
Ng and $A\beta_{1-42}$	0.089 [0.259 to 0.717]	0.435	1.000	80	0.078 [-0.0150 to 0.395]	0.219	1.000	249
SNAP25 and Tau PET	NA	NA	NA	NA	NA	NA	NA	NA
SNAP25 and $A\beta_{1-42}$	-0.147 [-0.423 to 0.153]	0.334	1.000	45	0.199 [0.015 to 0.395]	<0.001	0.341	85
$A\beta_{1-42}$ and Tau PET	0.247 [0.129 to 0.359]	<0.001	<0.001	258	0.437 [0.318 to 0.542]	<0.001	<0.001	203

Two-sided Pearson correlation coefficients (r) along with 95% confidence intervals in brackets, p-values, p-values adjusted for multiple comparisons (P_{adj}) and sample sizes for the correlation tests between synaptic (CSF Ng and SNAP25), neurodegeneration (HCV, CSF NfL, t-tau), and tau pathology (tau PET) biomarkers in cognitively unimpaired (CU) and impaired (CI) individuals in the ADNI cohort. HCV and amyloid-β ($A\beta_{1-42}$) were inverted. Not available (NA). CSF (cerebrospinal fluid). Total-tau (T-tau). Hippocampal volume (HCV). Neurofilament light chain protein (NfL). Neurogranin (Ng). Synaptosomal-associated protein 25 (SNAP25). Positron emission tomography (PET). Alzheimer's Disease Neuroimaging Initiative (ADNI).

Supplementary Table 5. Correlation matrix estimates for synaptic, neurodegeneration and tau pathology biomarkers in cognitively unimpaired individuals in the WRAP cohort.

**Biomarkers**	**Pearson r**	**P-value**	P_{adj}	**N**
HCV and NfL	0.114 [-0.005 to 0.231]	0.061	0.365	269
HCV and T-tau	<0.001[-0.119 to 0.120]	0.996	1.000	269
HCV and Ng	-0.020 [-0.139 to 0.0996]	0.741	1.000	269
HCV and SNAP25	0.017 [-0.145 to 0.178]	0.836	1.000	148
HCV and Tau PET	0.029 [-0.169 to 0.225]	0.776	1.000	99
HCV and $A\beta_{1-42}$	0.062 [-0.0576 to 0.181]	0.308	1.000	269
NfL and T-tau	0.484 [0.387 to 0.571]	<0.001	<0.001	269
NfL and Ng	0.377 [0.269 to 0.475]	<0.001	<0.001	269
NfL and SNAP25	0.459 [0.322 to 0.578]	<0.001	<0.001	148
NfL and Tau PET	0.035 [-0.164 to 0.231]	0.732	1.000	99
NfL and $A\beta_{1-42}$	-0.091 [-0.209 to 0.0287]	0.136	0.815	269
T-tau and Ng	0.923 [0.903 to 0.939]	<0.001	<0.001	269
T-tau and SNAP25	0.940 [0.918 to 0.956]	<0.001	<0.001	148
T-tau and Tau PET	0.194 [-0.00334 to 0.377]	0.054	0.325	99
T-tau and $A\beta_{1-42}$	-0.274 [-0.381 to -0.160]	<0.001	<0.001	269
Ng and SNAP25	0.888 [0.848 to 0.918]	<0.001	<0.001	148
Ng and Tau PET	0.179 [-0.0188 to 0.364]	0.076	0.454	99
Ng and $A\beta_{1-42}$	-0.372 [-0.470 to -0.264]	<0.001	<0.001	269
SNAP25 and Tau PET	0.175 [-0.0334 to 0.369]	0.099	0.595	90
SNAP25 and $A\beta_{1-42}$	-0.314 [-0.453 to -0.161]	<0.001	0.001	148
$A\beta_{1-42}$ and Tau PET	-0.073 [-0.267 to 0.126]	0.472	0.001	99

Two-sided Pearson correlation coefficients (r) along with 95% confidence intervals in brackets, p-values, p-values adjusted for multiple comparisons (P_{adj}) and sample sizes for the correlation tests between synaptic (CSF Ng and SNAP25), neurodegeneration (HCV, CSF NfL, t-tau), and tau pathology (tau PET) biomarkers in cognitively unimpaired (CU) individuals in the WRAP cohort. HCV and $A\beta_{1-42}$ were inverted. CSF (cerebrospinal fluid). Amyloid-β (Aβ). Total-tau (T-tau). Hippocampal volume (HCV). Neurofilament light chain protein (NfL). Neurogranin (Ng). Synaptosomal-associated protein 25 (SNAP25). Positron emission tomography (PET). Wisconsin Registry for Alzheimer's Prevention (WRAP).

Supplementary Figure 2 Associations of CSF t-tau with neurodegeneration and synaptic biomarkers in cognitively unimpaired and impaired individuals in two independent cohorts.

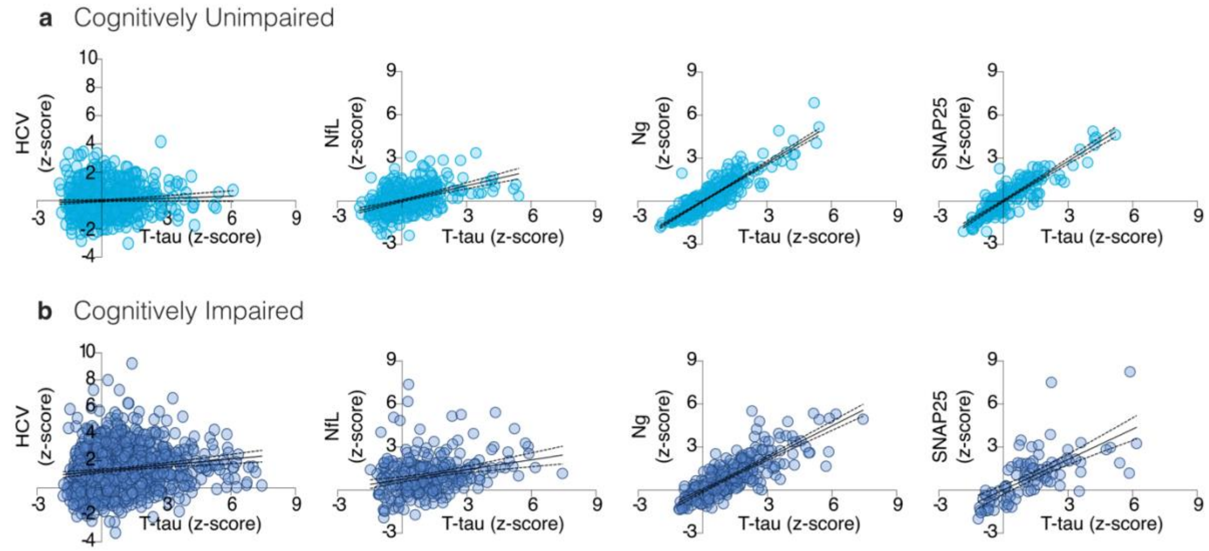

Scatter plots from linear regressions adjusted for age and sex, showing the association of CSF t-tau with biomarkers of established neurodegeneration (HCV, CSF NfL) and synaptic dysfunction (CSF Ng and SNAP25) in **(a)** CU and **(b)** CI individuals across two independent cohorts. Cognitively unimpaired (CU). Cognitively impaired (CI). CSF (cerebrospinal fluid). Total-tau (T-tau). Hippocampal volume (HCV). Neurofilament light chain protein (NfL). Neurogranin (Ng). Synaptosomal-associated protein 25 (SNAP25). Source data are provided as a Source Data file.

Supplementary Figure 3. Associations of CSF t-tau with neurodegeneration and synaptic biomarkers stratified by sex and cognition in two independent cohorts.

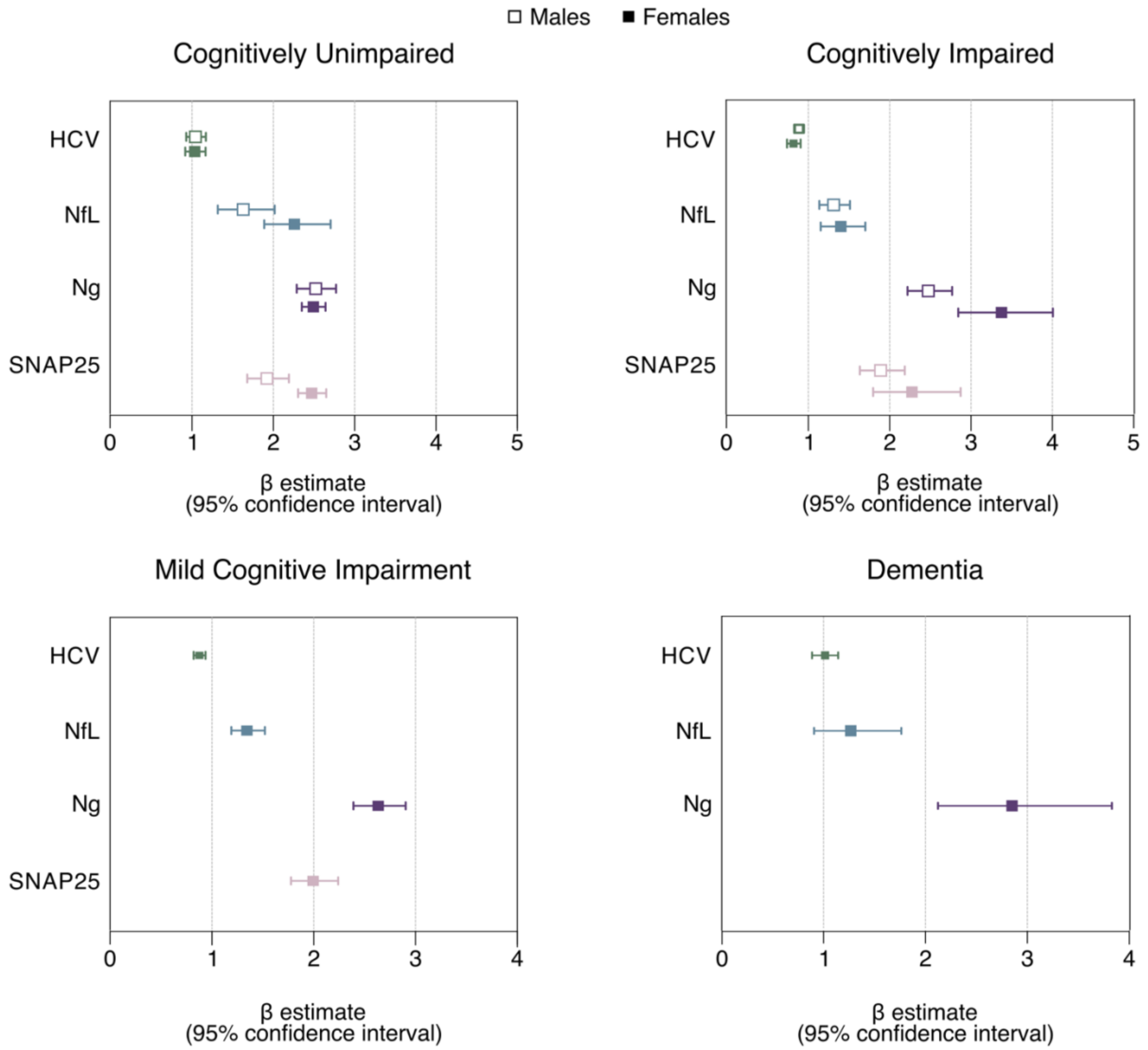

Mean β estimates with 95% confidence intervals from linear regressions, adjusted for age, cohort and sex where applicable, showing the association of CSF t-tau with biomarkers of established neurodegeneration (HCV, CSF NfL) and synaptic dysfunction (CSF Ng and SNAP25) stratified by cognitive status and sex **(a)** or by clinical diagnosis **(b)**, across cohorts. SNAP25 was not modeled in the dementia group due to limited sample size. CSF (cerebrospinal fluid). Total-tau (T-tau). Hippocampal volume (HCV). Neurofilament light chain protein (NfL). Neurogranin (Ng). Synaptosomal-associated protein 25 (SNAP25). Source data are provided as a Source Data file.

Supplementary Figure 4. Associations of CSF p-tau181 and plasma p-tau217 with synaptic biomarkers

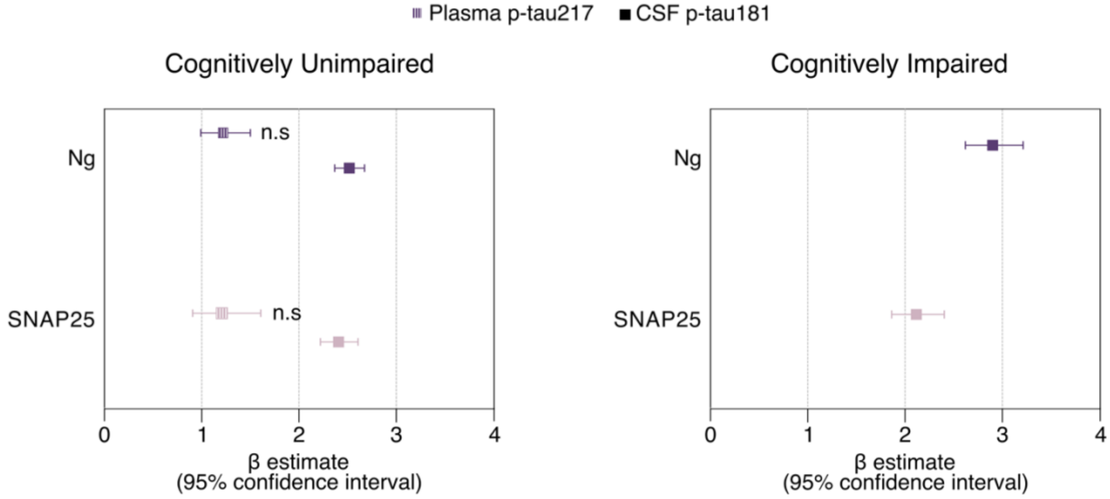

Mean β estimates with 95% confidence intervals from linear regressions, adjusted for age, sex and cohort where applicable, showing the association of synaptic dysfunction biomarkers (CSF Ng and SNAP25) and CSF p-tau181 across cohorts, and plasma p-tau217 (only in WRAP cohort). Neurogranin (Ng). Non-significant (n.s.). CSF (cerebrospinal fluid). Total-tau (T-tau). Neurogranin (Ng). Synaptosomal-associated protein 25 (SNAP25). Wisconsin Registry for Alzheimer's Prevention (WRAP). Source data are provided as a Source Data file.

Supplementary Figure 5 Associations of CSF t-tau with neurodegeneration and synaptic biomarkers in cognitively unimpaired and impaired individuals in two independent cohorts.

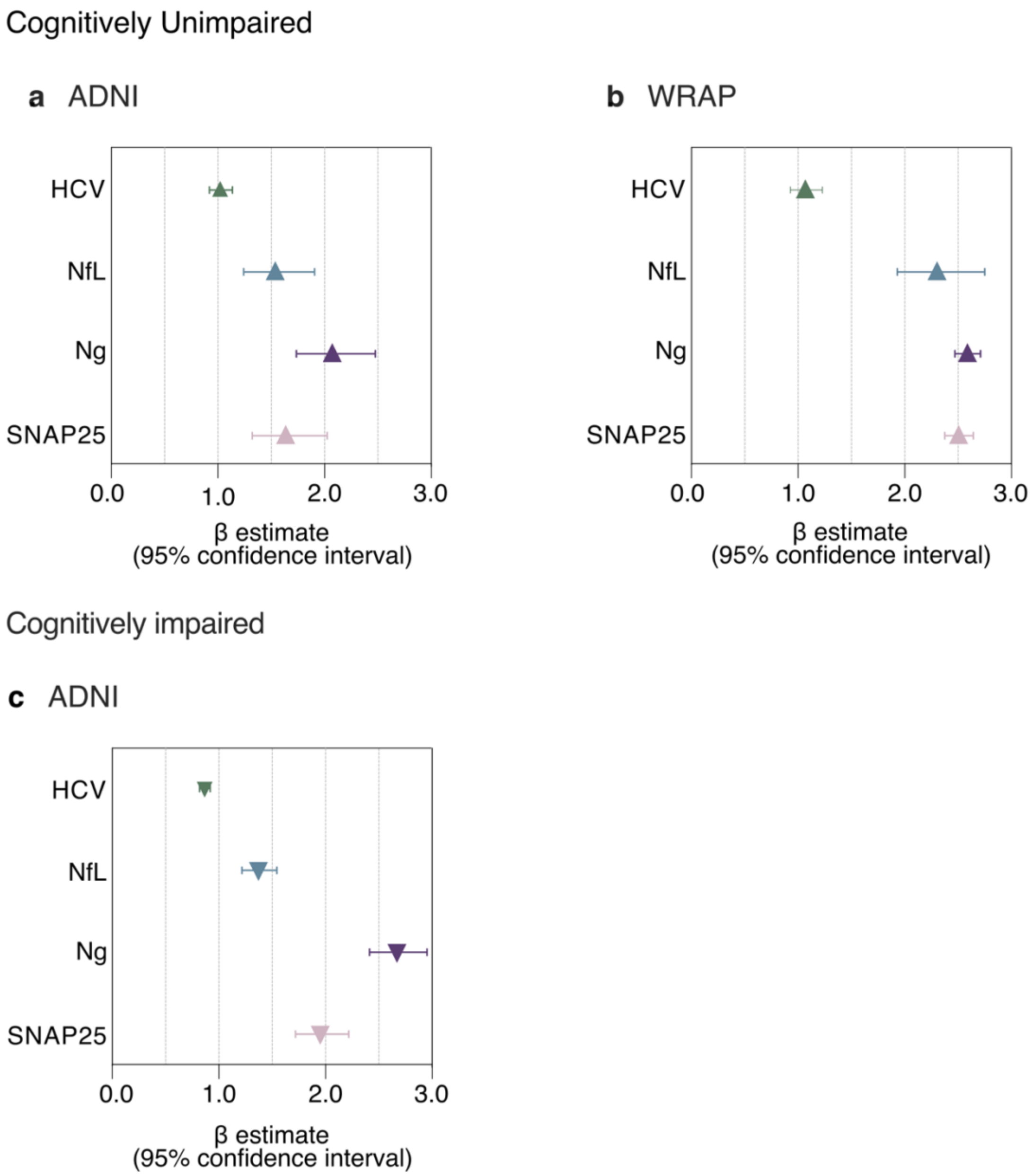

Mean β estimates with 95% confidence intervals from linear regressions, adjusted for age and sex, showing the association of CSF t-tau with biomarkers of established neurodegeneration (HCV, CSF NfL) and synaptic dysfunction (CSF Ng and SNAP25) in **(a-b)** CU in each cohort and **(c)** CI individuals in ADNI. Cognitively unimpaired (CU). Cognitively impaired (CI). Amyloid-β (Aβ). CSF (cerebrospinal fluid). Total-tau (T-tau). Hippocampal volume (HCV). Neurofilament light chain protein (NfL). Neurogranin (Ng). Synaptosomal-associated protein 25 (SNAP25). Cohorts: Alzheimer's Disease Neuroimaging Initiative (ADNI). Wisconsin Registry for Alzheimer's Prevention (WRAP). Source data are provided as a Source Data file.

Supplementary Table 6. Association of CSF t-tau with biomarkers of synaptic dysfunction and neurodegeneration in cognitively unimpaired individuals across two independent cohorts.

		95 % confidence interval				
Model	β	Lower	Upper	p-value	N	R^2
Model 1: HCV	1.038	0.955	1.128	0.384	733	0.072
Age	1.046	1.033	1.060	<0.001	733	0.072
Sex	0.828	0.701	0.977	0.026	733	0.072
Cohort	1.078	1.408	2.072	<0.001	733	0.072
Model 2: CSF NfL	1.979	1.724	2.269	<0.001	355	0.256
Age	0.985	0.967	1.003	0.105	355	0.256
Sex	0.681	0.548	0.846	0.001	355	0.256
Cohort	0.867	0.633	1.189	0.375	355	0.256
Model 3: CSF Ng	2.491	2.369	2.620	<0.001	349	0.797
Age	1.015	1.007	1.023	<0.001	349	0.797
Sex	1.148	1.026	1.285	0.016	349	0.797
Cohort	1.231	1.057	1.433	0.008	349	0.797
Model 4: CSF SNAP25	2.292	2.146	2.447	<0.001	193	0.783
Age	1.022	1.010	1.033	<0.001	193	0.783
Sex	0.951	0.811	1.116	0.539	193	0.783
Cohort	0.911	0.737	1.125	0.382	183	0.783

Mean β estimates, 95 % confidence interval, p-values and R^2 for the linear regressions testing the association between CSF t-tau and synaptic and neurodegeneration biomarkers, adjusted for age, sex, and cohort. Variables were log-transformed and z-scored. Amyloid-β (Aβ). Cerebrospinal fluid (CSF). Total-tau (T-tau). Hippocampal volume (HCV). Neurofilament light chain protein (NfL). Neurogranin (Ng). Synaptosomal-associated protein 25 (SNAP25). Cohorts: Alzheimer's Disease Neuroimaging Initiative (ADNI). Wisconsin Registry for Alzheimer's Prevention (WRAP).

Supplementary Table 7. Association of FDG-PET AD-ROI with biomarkers of synaptic dysfunction and neurodegeneration in cognitively unimpaired individuals in the ADNI cohort.

		95 % confidence interval				
Model	β	Lower	Upper	p-value	N	R^2
Model 1: CSF t-tau	1.100	0.951	1.272	0.198	226	0.093
Age	1.061	1.035	1.088	<0.001	226	0.093
Sex	0.857	0.637	1.154	0.309	226	0.093
Model 2: HCV	1.119	0.992	1.262	0.068	223	0.198
Age	0.935	0.916	0.954	<0.001	223	0.198
Sex	1.143	0.892	1.465	0.289	223	0.198
Model 3: CSF NfL	1.026	0.801	1.314	0.835	44	0.323
Age	1.116	1.053	1.183	<0.001	44	0.323
Sex	1.487	0.843	2.623	0.165	44	0.323
Model 4: CSF Ng	0.927	0.666	1.290	0.645	40	0.077
Age	1.039	0.965	1.118	0.303	40	0.077
Sex	1.369	0.680	2.757	0.369	40	0.077
Model 5: CSF SNAP25	0.732	0.465	1.154	0.167	23	0.158
Age	0.952	0.850	1.065	0.370	23	0.158
Sex	0.567	0.152	2.111	0.378	23	0.158

Mean β estimates, 95 % confidence interval, p-values and R^2 for the linear regressions testing the association between FDG-PET AD-ROI and synaptic and neurodegeneration biomarkers, adjusted for age and sex. Variables were log-transformed and z-scored. [^{18}F]Fluorodeoxyglucose positron emission tomography (FDG-PET). Amyloid-β (Aβ). Cerebrospinal fluid (CSF). Total-tau (T-tau). Hippocampal volume (HCV). Neurofilament light chain protein (NfL). Neurogranin (Ng). Synaptosomal-associated protein 25 (SNAP25). Alzheimer's Disease Neuroimaging Initiative (ADNI). Source data are provided as a Source Data file.

Supplementary Table 8. Association of CSF t-tau with biomarkers of synaptic dysfunction and neurodegeneration in cognitively unimpaired individuals across two independent cohorts.

Model	β	95 % confidence interval		p-value	N	R^2
		Lower conf. int.	Upper conf. int.			
**Females**						
Model 1: HCV	1.035	0.918	1.167	0.571	447	0.045
Age	1.035	1.018	1.053	<0.001	447	0.045
Cohort	1.600	1.253	2.044	<0.001	447	0.045
Model 2: CSF NfL	2.262	1.889	2.708	<0.001	226	0.289
Age	0.969	0.947	0.992	0.008	226	0.289
Cohort	0.667	0.430	1.034	0.070	226	0.289
Model 3: CSF Ng	2.495	2.352	2.645	<0.001	225	0.815
Age	1.006	0.996	1.016	0.242	225	0.815
Cohort	1.005	0.819	1.233	0.960	225	0.815
Model 4: CSF SNAP25	2.474	2.308	2.651	<0.001	124	0.854
Age	1.011	0.999	1.024	0.071	124	0.854
Cohort	0.885	0.684	1.145	0.349	124	0.854
**Males**						
Model 1: HCV	1.044	0.930	1.171	0.466	286	0.124
Age	1.064	1.043	1.086	<0.001	286	0.124
Cohort	1.928	1.409	2.639	<0.001	286	0.124
Model 2: CSF NfL	1.631	1.319	2.018	<0.001	129	0.223
Age	1.011	0.980	1.044	0.474	129	0.223
Cohort	1.214	0.768	1.920	0.404	129	0.223
Model 3: CSF Ng	2.521	2.291	2.773	<0.001	124	0.779
Age	1.032	1.017	1.046	<0.001	124	0.779
Cohort	1.636	1.303	2.053	<0.001	124	0.779
Model 4: CSF SNAP25	1.921	1.682	2.195	<0.001	69	0.653
Age	1.042	1.020	1.063	<0.001	69	0.653
Cohort	1.084	0.766	1.533	0.645	69	0.653

Mean β estimates, 95 % confidence interval, p-values and R^2 for the linear regressions testing the association between CSF t-tau and synaptic and neurodegeneration biomarkers, adjusted for age and cohort. Variables were log-transformed and z-scored. Amyloid-β (Aβ). Cerebrospinal fluid (CSF). Total-tau (T-tau). Hippocampal volume (HCV). Neurofilament light chain protein (NfL). Neurogranin (Ng). Synaptosomal-associated protein 25 (SNAP25). Cohorts: Alzheimer's Disease Neuroimaging Initiative (ADNI). Wisconsin Registry for Alzheimer's Prevention (WRAP).

Supplementary Table 9. Association of CSF p-tau181 and plasma p-tau217 with biomarkers of synaptic dysfunction in cognitively unimpaired and impaired individuals

Model	β	95 % confidence interval		p-value	N	R^2
		Lower conf. int.	Upper conf. int.			
**Cognitively unimpaired**						
Model 1: CSF p-tau181 and CSF Ng	2.517	2.369	2.675	<0.001	348	0.737
Age	1.019	1.010	1.029	<0.001	348	0.737
Sex	1.184	1.034	1.355	0.014	348	0.737
Cohort	1.177	0.981	1.413	0.079	348	0.737
Model 2: CSF p-tau181 and CSF SNAP25	2.407	2.221	2.608	<0.001	192	0.732
Age	1.025	1.012	1.039	<0.001	192	0.732
Sex	0.983	0.811	1.192	0.859	192	0.732
Cohort	0.895	0.694	1.154	0.389	192	0.732
Model 3: Plasma p-tau217 and CSF Ng	1.221	0.993	1.502	0.058	118	0.099
Age	1.031	0.994	1.069	0.106	118	0.099
Sex	1.664	1.013	2.733	0.044	118	0.099
Model 4: Plasma p-tau217 and CSF SNAP25	1.209	0.909	1.608	0.187	52	0.204
Age	1.069	1.009	1.132	0.025	52	0.204
Sex	1.926	0.918	4.041	0.082	52	0.204
**Cognitively impaired**						
Model 1: CSF p-tau181 and CSF Ng	2.896	2.616	3.207	<0.001	265	0.630
Age	1.012	0.999	1.025	0.070	265	0.630
Sex	1.100	0.895	1.353	0.363	265	0.630
Cohort	0.704	0.467	1.061	0.093	265	0.630
Model 2: CSF p-tau181 and CSF SNAP25	2.112	1.858	2.400	<0.001	93	0.652
Age	1.005	0.975	1.036	0.746	93	0.652
Sex	0.452	0.302	0.679	<0.001	93	0.652
Cohort	0.608	0.313	1.182	0.140	93	0.652

Mean β estimates, 95 % confidence interval, p-values and R^2 for the linear regressions testing the association between synaptic biomarkers and CSF p-tau181 (across cohorts) and plasma p-tau217 (WRAP cohort only) adjusted for age, sex, and cohort where applicable. Variables were log-transformed and z-scored. Amyloid-β (Aβ). Cerebrospinal fluid (CSF). Total-tau (T-tau). Hippocampal volume (HCV). Neurofilament light chain protein (NfL). Neurogranin (Ng). Synaptosomal-associated protein 25 (SNAP25). Cohorts: Alzheimer's Disease Neuroimaging Initiative (ADNI). Wisconsin Registry for Alzheimer's Prevention (WRAP).

Supplementary Table 10. Association of CSF t-tau with biomarkers of synaptic dysfunction and neurodegeneration in cognitively unimpaired and impaired individuals in the ADNI cohort.

		95 % confidence interval				
Model	β	Lower	Upper	p-value	N	R^2
**Cognitively unimpaired**						
Model 1: HCV	1.025	0.923	1.139	0.638	464	0.081
Age	1.054	1.036	1.072	<0.001	464	0.081
Sex	0.819	0.669	1.004	0.054	464	0.081
Model 2: CSF NfL	1.539	1.244	1.905	<0.001	86	0.248
Age	1.021	0.976	1.067	0.361	86	0.248
Sex	0.709	0.474	1.061	0.094	86	0.248
Model 3: CSF Ng	2.072	1.736	2.473	<0.001	80	0.528
Age	1.034	1.002	1.067	0.035	80	0.528
Sex	0.98	0.723	1.329	0.896	80	0.528
Model 4: CSF SNAP25	1.637	1.324	2.024	<0.001	45	0.378
Age	1.032	0.986	1.079	0.171	45	0.378
Sex	0.939	0.593	1.487	0.784	45	0.378
**Cognitively impaired**						
Model 1: HCV	0.867	0.819	0.919	<0.001	852	0.066
Age	1.012	0.999	1.025	0.072	852	0.066
Sex	0.621	0.515	0.748	<0.001	852	0.066
Model 2: CSF NfL	1.370	1.217	1.543	<0.001	257	0.120
Age	0.981	0.962	1.001	0.061	257	0.120
Sex	0.61	0.451	0.825	0.001	257	0.120
Model 3: CSF Ng	2.670	2.414	2.953	<0.001	249	0.609
Age	1.014	1.001	1.026	0.029	249	0.609
Sex	1.038	0.854	1.263	0.705	249	0.609
Model 4: CSF SNAP25	1.952	1.718	2.218	<0.001	85	0.637
Age	1.01	0.981	1.04	0.509	85	0.637
Sex	0.46	0.312	0.677	<0.001	85	0.637

Mean β estimates, 95 % confidence interval, p-values and R^2 for the linear regressions testing the association between CSF t-tau and synaptic and neurodegeneration biomarkers, adjusted for age, sex, and cohort. Variables were log-transformed and z-scored. Amyloid-β (Aβ). Cerebrospinal fluid (CSF). Total-tau (T-tau). Hippocampal volume (HCV). Neurofilament light chain protein (NfL). Neurogranin (Ng). Synaptosomal-associated protein 25 (SNAP25). Alzheimer's Disease Neuroimaging Initiative (ADNI).

Supplementary Table 11. Association of CSF t-tau with biomarkers of synaptic dysfunction and neurodegeneration in cognitively unimpaired individuals in the WRAP cohort.

		95 % confidence interval				
Model	β	Lower	Upper	p-value	N	R^2
**Cognitively unimpaired**						
Model 1: HCV	1.068	0.929	1.228	0.352	269	0.045
Age	1.036	1.015	1.057	0.001	269	0.045
Sex	0.841	0.627	1.126	0.243	269	0.045
Model 2: CSF NfL	2.304	1.930	2.751	<0.001	269	0.277
Age	0.972	0.952	0.993	0.008	269	0.277
Sex	0.682	0.528	0.881	0.004	269	0.277
Model 3: CSF Ng	2.588	2.471	2.711	<0.001	269	0.867
Age	1.012	1.004	1.019	0.002	269	0.867
Sex	1.241	1.111	1.386	<0.001	269	0.867
Model 4: CSF SNAP25	2.506	2.375	2.644	<0.001	148	0.896
Age	1.018	1.009	1.027	<0.001	148	0.896
Sex	0.927	0.809	1.062	0.27	148	0.896

Mean β estimates, 95 % confidence interval, p-values and R^2 for the linear regressions testing the association between CSF t-tau and synaptic and neurodegeneration biomarkers, adjusted for age, sex, and cohort. Variables were log-transformed and z-scored. Amyloid-β (Aβ). Cerebrospinal fluid (CSF). Total-tau (T-tau). Hippocampal volume (HCV). Neurofilament light chain protein (NfL). Neurogranin (Ng). Synaptosomal-associated protein 25 (SNAP25). Registry for Alzheimer's Prevention (WRAP).

Supplementary Table 12. Association of CSF t-tau with biomarkers of synaptic dysfunction and neurodegeneration in cognitively impaired individuals across two independent cohorts.

		95 % confidence interval				
Model	β	Lower	Upper	p-value	N	R^2
Model 1: HCV	0.868	0.819	0.919	<0.001	868	0.066
Age	1.1013	1.000	1.026	0.059	868	0.066
Sex	0.623	0.517	0.750	<0.001	868	0.066
Cohort	0.986	0.504	1.928	0.967	868	0.066
Model 2: CSF NfL	1.400	1.244	1.576	<0.001	273	0.128
Age	0.982	0.963	1.002	0.073	273	0.128
Sex	0.602	0.447	0.809	0.001	273	0.128
Cohort	0.594	0.325	1.085	0.090	273	0.128
Model 3: CSF Ng	2.669	2.434	2.027	<0.001	265	0.638
Age	1.013	1.002	1.025	0.026	265	0.638
Sex	1.072	0.889	1.292	0.465	265	0.638
Cohort	0.880	0.607	1.275	0.498	265	0.638
Model 4: CSF SNAP25	2.020	1.798	2.270	<0.001	93	0.665
Age	1.011	0.983	1.039	0.457	93	0.665
Sex	0.476	0.329	0.689	<0.001	93	0.665
Cohort	0.762	0.416	1.393	0.372	93	0.665

Mean β estimates, 95 % confidence interval, p-values and R^2 for the linear regressions testing the association between CSF t-tau and synaptic and neurodegeneration biomarkers, adjusted for age, sex, and cohort. Variables were log-transformed and z-scored. Amyloid-β (Aβ). Cerebrospinal fluid (CSF). Total-tau (T-tau). Hippocampal volume (HCV). Neurofilament light chain protein (NfL). Neurogranin (Ng). Synaptosomal-associated protein 25 (SNAP25). Cohorts: Alzheimer's Disease Neuroimaging Initiative (ADNI). Wisconsin Registry for Alzheimer's Prevention (WRAP).

Supplementary Table 13. Association of FDG-PET AD-ROI with biomarkers of synaptic dysfunction and neurodegeneration in cognitively impaired individuals in the ADNI cohort.

		95 % confidence interval				
Model	β	Lower	Upper	p-value	N	R^2
Model 1: CSF t-tau	0.760	0.713	0.810	<0.001	694	0.126
Age	1.021	1.008	1.034	0.002	694	0.126
Sex	0.589	0.481	0.722	<0.001	694	0.126
Model 2: HCV	1.508	1.406	1.618	<0.001	687	0.294
Age	0.918	0.905	0.931	<0.001	687	0.294
Sex	1.168	0.933	1.461	0.175	687	0.294
Model 3: CSF NfL	0.816	0.701	0.951	0.009	120	0.195
Age	1.053	1.022	1.084	0.001	120	0.195
Sex	1.680	1.073	2.630	0.024	120	0.195
Model 4: CSF Ng	1.048	0.936	1.172	0.413	117	0.082
Age	0.976	0.955	0.997	0.028	117	0.082
Sex	0.764	0.548	1.065	0.111	117	0.082
Model 5: CSF SNAP25	1.130	0.808	1.580	0.465	44	0.089
Age	0.943	0.874	1.016	0.121	44	0.089
Sex	0.889	0.346	2.285	0.802	44	0.089

Mean β estimates, 95 % confidence interval, p-values and R^2 for the linear regressions testing the association between FDG-PET AD-ROI and synaptic and neurodegeneration biomarkers, adjusted for age and sex. Variables were log-transformed and z-scored. [^{18}F]Fluorodeoxyglucose positron emission tomography (FDG-PET). Amyloid-β (Aβ). Cerebrospinal fluid (CSF). Total-tau (T-tau). Hippocampal volume (HCV). Neurofilament light chain protein (NfL). Neurogranin (Ng). Synaptosomal-associated protein 25 (SNAP25). Cohorts: Alzheimer's Disease Neuroimaging Initiative (ADNI). Source data are provided as a Source Data file.

Supplementary Table 14. Association of CSF t-tau with biomarkers of synaptic dysfunction and neurodegeneration in cognitively impaired individuals across two independent cohorts.

Model	β	95 % confidence interval		p-value	N	R^2
		Lower	Upper			
**Females**						
Model 1: HCV	0.826	0.746	0.914	<0.001	359	0.050
Age	1.005	0.985	1.026	0.619	359	0.050
Cohort	0.928	0.391	2.202	0.865	359	0.050
Model 2: CSF NfL	1.407	1.159	1.707	0.001	107	0.121
Age	0.975	0.947	1.003	0.082	107	0.121
Cohort	0.601	0.302	1.193	0.144	107	0.121
Model 3: CSF Ng	3.372	2.843	4.001	<0.001	106	0.666
Age	1.021	1.004	1.039	0.019	106	0.666
Cohort	0.762	0.502	1.157	0.200	106	0.666
Model 4: CSF SNAP25	2.275	1.802	2.872	<0.001	36	0.622
Age	1.001	0.954	1.051	0.959	36	0.622
Cohort	0.728	0.363	1.459	0.359	36	0.622
**Males**						
Model 1: HCV	0.894	0.835	0.958	0.001	509	0.046
Age	1.018	1.001	1.035	0.033	509	0.046
Cohort	1.178	0.379	3.664	0.777	509	0.046
Model 2: CSF NfL	1.318	1.143	1.520	<0.001	167	0.085
Age	0.991	0.965	1.018	0.520	167	0.085
Cohort	0.639	0.209	1.953	0.430	167	0.085
Model 3: CSF Ng	2.479	2.221	2.767	<0.001	159	0.634
Age	1.012	0.996	1.028	0.144	159	0.634
Cohort	1.236	0.634	2.408	0.532	159	0.634
Model 4: CSF SNAP25	1.895	1.640	2.190	<0.001	57	0.646
Age	1.013	0.977	1.049	0.483	57	0.646
Cohort	1.596	0.313	8.128	0.567	57	0.646

Mean β estimates, 95 % confidence interval, p-values and R^2 for the linear regressions testing the association between CSF t-tau and synaptic and neurodegeneration biomarkers, adjusted for age and cohort. Variables were log-transformed and z-scored. Amyloid-β (Aβ). Cerebrospinal fluid (CSF). Total-tau (T-tau). Hippocampal volume (HCV). Neurofilament light chain protein (NfL). Neurogranin (Ng). Synaptosomal-associated protein 25 (SNAP25). Cohorts: Alzheimer's Disease Neuroimaging Initiative (ADNI). Wisconsin Registry for Alzheimer's Prevention (WRAP).

Supplementary Table 15. Association of CSF t-tau with biomarkers of synaptic dysfunction and neurodegeneration in individuals with mild cognitive impairment or dementia across two independent cohorts.

Model	β	95 % confidence interval		p-value	N	R^2
		Lower	Upper			
**MCI**						
Model 1: HCV	0.878	0.821	0.939	<0.001	736	0.065
Age	1.021	1.006	1.036	0.006	736	0.065
Sex	0.641	0.523	0.787	<0.001	736	0.065
Cohort	1.071	0.543	2.114	0.842	736	0.065
Model 2: CSF NfL	1.346	1.191	1.522	<0.001	234	0.114
Age	0.990	0.968	1.013	0.392	234	0.114
Sex	0.587	0.421	0.820	0.002	234	0.114
Cohort	0.622	0.334	1.156	0.132	234	0.114
Model 3: CSF Ng	2.635	2.391	2.905	<0.001	225	0.645
Age	1.017	1.004	1.030	0.012	225	0.645
Sex	1.084	0.881	1.334	0.444	225	0.645
Cohort	0.920	0.633	1.336	0.659	225	0.645
Model 4: CSF SNAP25	1.996	1.778	2.241	<0.001	87	0.672
Age	1.007	0.977	1.037	0.653	87	0.672
Sex	0.513	0.351	0.750	0.001	87	0.672
Cohort	0.833	0.459	1.514	0.545	87	0.672
**Dementia**						
Model 1: HCV	1.008	0.887	1.146	0.899	132	0.063
Age	0.986	0.962	1.010	0.257	132	0.063
Sex	0.624	0.417	0.934	0.022	132	0.063
Model 2: CSF NfL	1.267	0.909	1.765	0.157	40	0.116
Age	0.962	0.922	1.005	0.080	40	0.116
Sex	0.726	0.335	1.572	0.406	40	0.116
Model 3: CSF Ng	2.853	2.126	3.830	<0.001	40	0.618
Age	0.999	0.971	1.028	0.946	40	0.618
Sex	1.124	0.690	1.828	0.631	40	0.618

β estimates, 95 % confidence interval, p-values and R^2 for the linear regressions testing the association between CSF t-tau and synaptic and neurodegeneration biomarkers, adjusted for age, sex and cohort where applicable. WRAP cohort did not include individuals with dementia. Variables were log-transformed and z-scored. Mild cognitive impairment (MCI). Amyloid-β (Aβ). Cerebrospinal fluid (CSF). Total-tau (T-tau). Hippocampal volume (HCV). Neurofilament light chain protein (NfL). Neurogranin (Ng). Synaptosomal-associated protein 25 (SNAP25). Cohorts: Alzheimer's Disease Neuroimaging Initiative (ADNI). Wisconsin Registry for Alzheimer's Prevention (WRAP).

Supplementary Table 16. Association of CSF t-tau with biomarkers of synaptic dysfunction and neurodegeneration in cognitively unimpaired and impaired individuals in the ADNI cohort.

Model	pR^2	pR^2 (%)	ΔpR^2	R^2	AIC
**Cognitively unimpaired**					
Full	-	-	-	0.67	67[a]
Neurodegeneration	0.17 ± 0.14	13.5 %	0.50	0.16	101[b]
Synapse degeneration	0.60 ± 0.11	87.1 %	-0.50	0.62	70
**Cognitively impaired**					
Full	-	-	-	0.80	136[a,b]
Neurodegeneration	0.23 ± 0.08	25.6 %	0.50	0.30	228[b]
Synapse degeneration	0.73 ± 0.05	88.4 %	-0.50	0.74	152

Proportion of variation of CSF t-tau levels explained by neurodegeneration (N) (HCV and CSF NfL combined) and synaptic degeneration (S) (Ng and SNAP25 combined) were estimated using partial R^2 (pR^2) from multivariable linear regression models adjusted for age, sex and cohort in cognitively unimpaired (n =40) and impaired (n = 76) groups. pR^2 values are reported as mean ± s.d. obtained by bootstrapping (n = 1000 iterations). Percentual pR^2 was calculated as the pR^2 of each model divided by the total R^2 of the model (100* pR^2/R^2). Percentage of pR^2 does not sum to 100% due to shared variability. pR^2 models were calculated based on full model (t-tau ~ HCV + NfL + Ng + SNAP25); and without the variables for S (t-tau ~ HCV + NfL) and N (t-tau ~ Ng + SNAP25) models. ΔpR^2 indicates difference between N and S pR^2 values. AIC values were calculated for models assessing t-tau levels associated with biomarkers of N and S. [a]AIC > 15 compared with N. [b]AIC > 15 compared with S. Akaike Information Criterion (AIC). Amyloid-β (Aβ). Cerebrospinal fluid (CSF). Total-tau (T-tau). Hippocampal volume (HCV). Neurofilament light chain protein (NfL). Neurogranin (Ng). Synaptosomal-associated protein 25 (SNAP25). Alzheimer's Disease Neuroimaging Initiative (ADNI). Standard deviation (s.d.).

Supplementary Table 17. Association of CSF t-tau with biomarkers of synaptic dysfunction and neurodegeneration in cognitively unimpaired individuals in the WRAP cohort.

Model	pR^2	pR^2 (%)	ΔpR^2	R^2	AIC
Full	-	-	-	0.94	45
Neurodegeneration	0.10 ± 0.04	10.0 %	0.82	0.32	417[a]
Synapse dysfunction	0.91 ± 0.02	96.7 %	-0.82	0.93	79[b]

Proportion of variation of CSF t-tau levels explained by neurodegeneration (N) (HCV and CSF NfL combined) and synaptic degeneration (S) (Ng and SNAP25 combined) were estimated using partial R^2 (pR^2) from multivariable linear regression models adjusted for age, sex and cohort in cognitively unimpaired (n = 148) group. pR^2 values are reported as mean ± s.d. obtained by bootstrapping (n = 1000 iterations). Percentual pR^2 was calculated as the pR^2 of each model divided by the total R^2 of the model ($100 * pR^2/R^2$). Percentage of pR^2 does not sum to 100% due to shared variability. pR^2 models were calculated based on full model (t-tau ~ HCV + NfL + Ng + SNAP25); and without the variables for S (t-tau ~ HCV + NfL) and N (t-tau ~ Ng + SNAP25) models. ΔpR^2 indicates difference between N and S pR^2 values. AIC values were calculated for models assessing t-tau levels associated with biomarkers of N and S. [a]AIC > 15 compared with N. [b]AIC > 15 compared with S. Akaike Information Criterion (AIC). Amyloid-β (Aβ). Cerebrospinal fluid (CSF). Total-tau (T-tau). Hippocampal volume (HCV). Neurofilament light chain protein (NfL). Neurogranin (Ng). Synaptosomal-associated protein 25 (SNAP25). Wisconsin Registry for Alzheimer's Prevention (WRAP). Standard deviation (s.d.).

Supplementary Table 18. Association of CSF p-tau181 with biomarkers of synaptic dysfunction and neurodegeneration in cognitively unimpaired and impaired individuals in two independent cohorts.

Model	pR^2	pR^2 (%)	ΔpR^2	R^2	AIC
**Cognitively unimpaired**					
Full	-	-	-	0.83	274[a]
Neurodegeneration	0.07	8.7 %	0.69	0.28	547[b]
Synapse degeneration	0.77	92.0 %	-0.69	0.82	286
**Cognitively impaired**					
Full	-	-	-	0.81	167[a]
Neurodegeneration	0.14	17.0 %	0.61	0.20	284[b]
Synapse degeneration	0.75	93.4 %	-0.61	0.77	177

Proportion of variation of CSF p-tau181 levels explained by neurodegeneration (N) (HCV and CSF NfL combined) and synaptic degeneration (S) (Ng and SNAP25 combined) were estimated using partial R^2 (pR^2) from multivariable linear regression models adjusted for age, sex and cohort in cognitively unimpaired (n =40) and impaired (n = 76) groups. Percentual pR^2 was calculated as the pR^2 of each model divided by the total R^2 of the model (100* pR^2/R^2). Percentage of pR^2 does not sum to 100% due to shared variability. pR^2 models were calculated based on full model (t-tau ~ HCV + NfL + Ng + SNAP25); and without the variables for S (p-tau181 ~ HCV + NfL) and N (t-tau ~ Ng + SNAP25) models. ΔpR^2 indicates difference between N and S pR^2 values. AIC values were calculated for models assessing t-tau levels associated with biomarkers of N and S. [a]AIC > 15 compared with N. [b]AIC > 15 compared with S. Akaike Information Criterion (AIC). Amyloid-β (Aβ). Cerebrospinal fluid (CSF). Total-tau (T-tau). Hippocampal volume (HCV). Neurofilament light chain protein (NfL). Neurogranin (Ng). Synaptosomal-associated protein 25 (SNAP25). Cohorts: Alzheimer's Disease Neuroimaging Initiative (ADNI). Wisconsin Registry for Alzheimer's Prevention (WRAP).

Supplementary Figure 6. CSF t-tau is increased in individuals with abnormal synaptic degeneration regardless of concomitant presence of neurodegeneration.

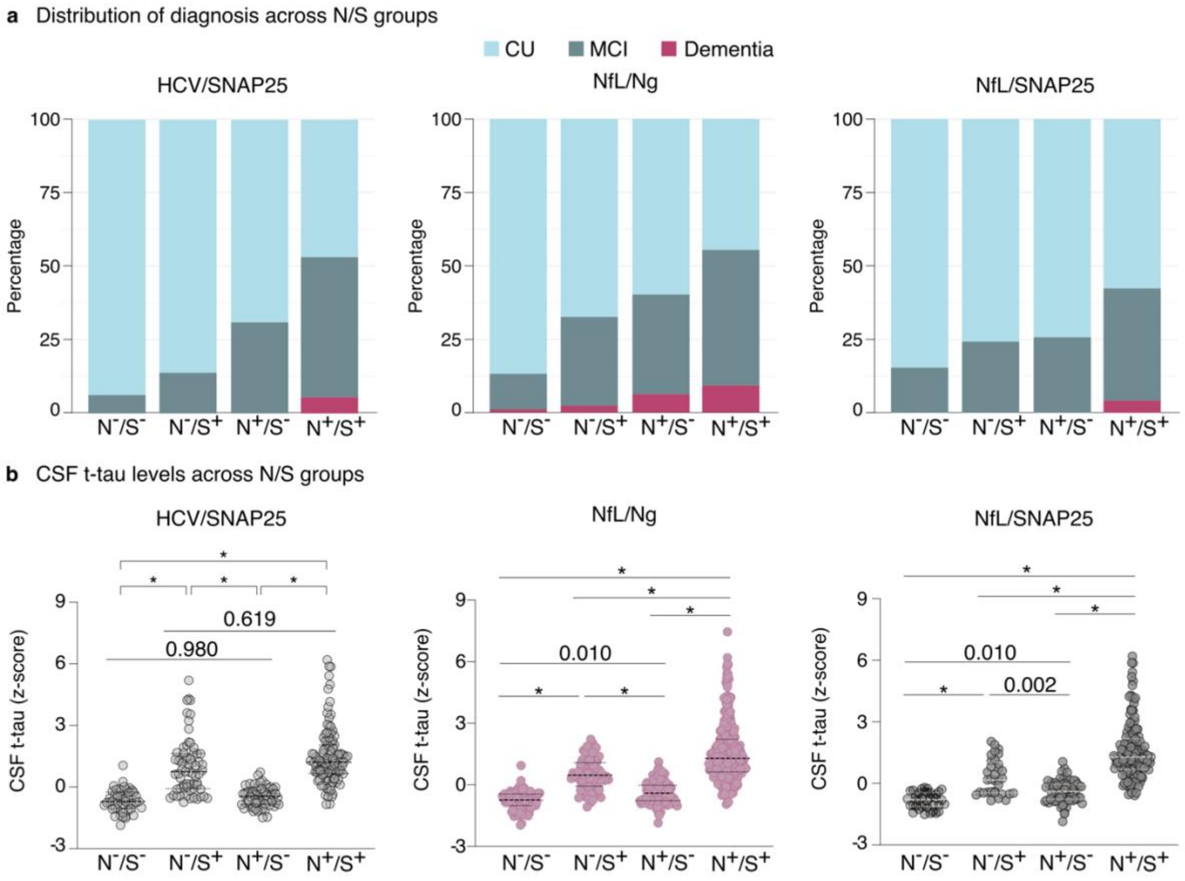

(**a**) Bar graphs show the distribution of CU, MCI and dementia across synaptic degeneration (S) and neurodegeneration (N) groups. N positivity was based on HCV or CSF NfL and S positivity was based on CSF Ng (n = 584) or SNAP25 (n = 280). Cutoffs were calculated as above or below median from CU Aβ^- individuals. (**b**) Violin plots show CSF t-tau levels in individuals with abnormal S and/or N in the whole population (CU and CI) across all cohorts. The median is shown by the middle dashed line, and the quartiles by the top and bottom dashed lines. CSF t-tau levels were compared across groups using a linear regression model with dummy variables, adjusted for age, sex, cognitive status, amyloid burden, and cohort. Pairwise comparisons were corrected for multiple testing using Tukey's method. * Padj-value < 0.0001. Amyloid-β (Aβ). Cognitively unimpaired (CU). Mild cognitive impairment (MCI). Neurodegeneration (N). Neurofilament light protein (NfL). Neurogranin (Ng). Synaptic degeneration (S). CSF (cerebrospinal fluid). Total-tau (T-tau). Synaptosomal-associated protein 25 (SNAP25). Cohorts: Alzheimer's Disease Neuroimaging Initiative (ADNI). Wisconsin Registry for Alzheimer's Prevention (WRAP). Source data are provided as a Source Data file.

Supplementary Table 19. CSF t-tau levels across N/S (HCV/Ng) profiles across two independent cohorts

Group reported	Compared to	β	SE	T-value	P-value	Padj-value
N-S+	N-S-	4.555	1.124	12.955	<0.0001	<0.0001
N+S-	N-S-	0.912	1.133	-0.739	0.461	0.882
N+S+	N-S-	5.113	1.123	14.099	<0.0001	<0.0001
N+S-	N-S+	0.200	1.119	-14.262	<0.0001	<0.0001
N+S+	N-S+	1.123	1.104	1.171	0.244	0.648
N+S+	N+S-	5.608	1.105	17.261	<0.0001	<0.0001
**Covariates**						
Age		1.012	1.005	2.371	0.018	-
Sex		1.018	1.077	0.237	0.813	-
Cohort		1.335	1.121	2.527	0.012	-
Cognitive status		1.935	1.115	6.052	<0.0001	-
Aβ burden		0.979	1.026	-0.828	0.408	-

Mean difference values of log-transformed and z-scored levels of t-tau comparing S and N profiles: N-S- (n = 85), N-S+ (n = 137), N+S- (n = 110), S+N+ (n = 252). Ng and HCV levels above median of CU A- individuals were used to classify individuals with abnormal synaptic dysfunction (S+) and abnormal neurodegeneration (N+). Mean differences were estimated with linear regression models adjusted for age, sex, cognitive status, amyloid burden (CSF $A\beta_{1-42}$), and cohort. Groups were added as dummy variables to assess differences in t-tau levels between groups, and pairwise comparisons were corrected for multiple testing using Tukey's method (Padj-value). A+ individuals were classified within each cohort according to previously published reference values (see Methods). Amyloid-β (A). Cognitively unimpaired (CU). Hippocampal volume (HCV). Neurogranin (Ng). Standard error (SE). CSF (cerebrospinal fluid). Total-tau (T-tau). Cohorts: Alzheimer's Disease Neuroimaging Initiative (ADNI). Wisconsin Registry for Alzheimer's Prevention (WRAP).

Supplementary Table 20 CSF t-tau levels across N/S (HCV/ SNAP25) profiles across two independent cohorts

Group reported	Compared to	β	SE	T-value	P-value	Padj-value
N-S+	N-S-	4.923	1.176	9.852	<0.001	<0.0001
N+S-	N-S-	1.068	1.186	0.384	0.702	0.980
N+S+	N-S-	5.865	1.175	10.998	<0.001	<0.0001
N+S-	N-S+	0.217	1.171	-9.706	<0.001	<0.0001
N+S+	N-S+	1.191	1.155	1.216	0.225	0.619
N+S+	N+S-	5.494	1.150	12.153	<0.001	<0.0001
**Covariates**						
Age		1.016	1.008	2.025	0.044	-
Sex		0.728	1.116	-2.901	0.004	-
Cohort		1.047	1.171	0.288	0.774	-
Cognitive status		1.469	1.178	2.349	0.020	-
Aβ burden		0.947	1.038	-1.442	0.151	-

Mean difference values of levels of t-tau comparing S and N profiles: N-S- (n = 48), N-S+(n = 65), N+S- (n = 58), N+S+ (n = 109). SNAP25 and HCV levels above median of CU A- individuals were used to classify individuals with abnormal synaptic dysfunction (S+) and abnormal neurodegeneration (N+). Mean differences were estimated with linear regression models adjusted for age, sex, cognitive status, amyloid burden (CSF $A\beta_{1-42}$), and cohort. Groups were added as dummy variables to assess differences in t-tau levels between groups, and pairwise comparisons were corrected for multiple testing using Tukey's method (Padj-value). A+ individuals were classified within each cohort according to previously published reference values (see Methods). Amyloid-β (A). Cognitively unimpaired (CU). Hippocampal volume (HCV). Standard error (SE). CSF (cerebrospinal fluid). Total-tau (T-tau). Synaptosomal-associated protein 25 (SNAP25). Cohorts: Alzheimer's Disease Neuroimaging Initiative (ADNI). Wisconsin Registry for Alzheimer's Prevention (WRAP).

Supplementary Table 21. CSF t-tau levels across N/S (NfL/Ng) profiles across two independent cohorts

Group reported	Compared to	β	SE	T-value	P-value	Padj-value
N-S+	N-S-	3.702	1.133	10.479	<0.001	<0.0001
N+S-	N-S-	1.471	1.130	3.157	0.002	0.010
N+S+	N-S-	7.426	1.110	19.139	<0.001	<0.0001
N+S-	N-S+	0.270	1.133	-10.479	<0.001	<0.0001
N+S+	N-S+	0.397	1.137	-7.189	<0.001	<0.0001
N+S+	N+S-	2.006	1.113	6.490	<0.001	<0.0001
**Covariates**						
Age		1.001	1.005	0.217	0.828	-
Sex		0.923	1.076	-1.097	0.273	-
Cohort		1.148	1.116	1.260	0.208	-
Cognitive status		1.768	1.108	5.544	<0.001	-
Aβ burden		0.984	1.024	-0.649	0.516	-

Mean difference values of levels of t-tau comparing S and N profiles: N-S- (n = 90), N-S+(n = 84), N+S- (n = 105), N+S+ (n = 305). CSF Ng and NfL levels above median of CU A- individuals were used to classify individuals with abnormal synaptic dysfunction (S+) and abnormal neurodegeneration (N+). Mean differences were estimated with linear regression models adjusted for age, sex, cognitive status, amyloid burden (CSF $A\beta_{1-42}$), and cohort. Groups were added as dummy variables to assess differences in t-tau levels between groups, and pairwise comparisons were corrected for multiple testing using Tukey's method (Padj-value). A+ individuals were classified within each cohort according to previously published reference values (see Methods). Amyloid-β (A). Cognitively unimpaired (CU). Standard error (SE). CSF (cerebrospinal fluid). Total-tau (T-tau). Neurogranin (Ng). Neurofilament light chain protein (NfL). Cohorts: Alzheimer's Disease Neuroimaging Initiative (ADNI). Wisconsin Registry for Alzheimer's Prevention (WRAP).

Supplementary Table 22. CSF t-tau levels across N/S (NfL/SNAP25) profiles across two independent cohorts

Group reported	Compared to	β	SE	T-value	P-value	Padj-value
N-S+	N-S-	3.214	1.208	6.168	<0.0001	<0.0001
N+S-	N-S-	1.698	1.183	3.147	0.002	0.010
N+S+	N-S-	9.125	1.164	14.536	<0.0001	<0.0001
N+S-	N-S+	0.311	1.208	-6.168	<0.0001	0.002
N+S+	N-S+	0.528	1.193	-3.616	<0.0001	<0.0001
N+S+	N+S-	2.839	1.176	6.436	<0.0001	<0.0001
**Covariates**						
Age		0.998	1.008	-0.237	0.813	-
Sex		0.695	1.107	-3.586	<0.0001	-
Cohort		0.815	1.162	-1.364	0.174	-
Cognitive status		1.392	1.158	2.259	0.025	-
Aβ burden		0.972	1.036	-0.809	0.419	-

Mean difference values of levels of t-tau comparing S and N profiles: N-S- (n = 38), N-S+(n = 33), N+S- (n = 68), N+S+ (n = 141). CSF SNAP25 and NfL levels above median of CU A- individuals were used to classify individuals with abnormal synaptic dysfunction (S+) and abnormal neurodegeneration (N+). Mean differences were estimated with linear regression models adjusted for age, sex, cognitive status, amyloid burden (CSF $A\beta_{1-42}$), and cohort. Groups were added as dummy variables to assess differences in t-tau levels between groups, and pairwise comparisons were corrected for multiple testing using Tukey's method (Padj-value). A+ individuals were classified within each cohort according to previously published reference values (see Methods). Amyloid-β (A). Cognitively unimpaired (CU). Standard error (SE). CSF (cerebrospinal fluid). Total-tau (T-tau). Synaptosomal-associated protein 25 (SNAP25). Neurofilament light chain protein (NfL). Cohorts: Alzheimer's Disease Neuroimaging Initiative (ADNI). Wisconsin Registry for Alzheimer's Prevention (WRAP).

Supplementary Table 23. CSF t-tau levels across N/S (HCV/Ng) profiles in ADNI cohort

Group reported	Compared to	β	SE	T-value	P-value	Padj-value
N-S+	N-S-	3.383	1.223	6.060	<0.0001	<0.0001
N+S-	N-S-	0.799	1.258	-0.977	0.329	0.763
N+S+	N-S-	4.544	1.223	7.530	<0.0001	<0.0001
N+S-	N-S+	0.236	1.208	-7.637	<0.0001	<0.0001
N+S+	N-S+	1.343	1.159	2.000	0.046	0.190
N+S+	N+S-	1.252	1.258	11.331	<0.0001	<0.0001
**Covariates**						
Age		1.004	1.008	0.509	0.611	-
Sex		0.911	1.111	-0.889	0.375	-
Cognitive status		1.844	1.151	4.354	<0.001	-
Aβ burden		0.945	1.034	-1.689	0.092	-

Mean difference values of levels of t-tau comparing S and N profiles: N-S- (n = 30), N-S+ (n = 57), N+S- (n = 42), N+S+ (n = 170). Ng and HCV levels above median of CU A- individuals were used to classify individuals with abnormal synaptic dysfunction (S+) and abnormal neurodegeneration (N+). Mean differences were estimated with linear regression models adjusted for age, sex, cognitive status, amyloid burden (CSF $A\beta_{1-42}$), and cohort. Groups were added as dummy variables to assess differences in t-tau levels between groups, and pairwise comparisons were corrected for multiple testing using Tukey's method (Padj-value). A+ individuals were classified within each cohort according to previously published reference values (see Methods). Amyloid-β (A). Cognitively unimpaired (CU). Hippocampal volume (HCV). Neurogranin (Ng). Standard error (SE). CSF (cerebrospinal fluid). Total-tau (T-tau). Synaptosomal-associated protein 25 (SNAP25). Alzheimer's Disease Neuroimaging Initiative (ADNI).

Supplementary Table 24. CSF t-tau levels across N/S (HCV/SNAP25) profiles in ADNI cohort

Group reported	Compared to	β	SE	T-value	P-value	Padj-value
N-S+	N-S-	3.112	1.347	3.814	<0.0001	0.001
N+S-	N-S-	0.997	1.359	-0.010	0.992	1.000
N+S+	N-S-	5.258	1.337	5.718	<0.0001	<0.0001
N+S-	N-S+	0.320	1.301	-4.329	<0.0001	0.0002
N+S+	N-S+	1.690	1.266	2.226	0.028	<0.122
N+S+	N+S-	5.274	1.234	7.923	<0.0001	<0.0001
**Covariates**						
Age		0.993	1.015	-0.508	0.612	-
Sex		0.661	1.186	-2.432	0.017	-
Cognitive status		1.263	1.231	1.12	0.265	-
Aβ burden		0.947	1.052	-1.071	0.287	-

Mean difference values of levels of t-tau comparing S and N profiles: N-S- (n = 14), N-S+ (n = 23), N+S- (n = 27), N+S+ (n = 60). SNAP25 and HCV levels above median of CU A- individuals were used to classify individuals with abnormal synaptic dysfunction (S+) and abnormal neurodegeneration (N+). Mean differences were estimated with linear regression models adjusted for age, sex, cognitive status, amyloid burden (CSF $A\beta_{1-42}$), and cohort. Groups were added as dummy variables to assess differences in t-tau levels between groups, and pairwise comparisons were corrected for multiple testing using Tukey's method (Padj-value). A+ individuals were classified within each cohort according to previously published reference values (see Methods). Amyloid-β (A). Cognitively unimpaired (CU). Hippocampal volume (HCV). Standard error (SE). CSF (cerebrospinal fluid). Total-tau (T-tau). Synaptosomal-associated protein 25 (SNAP25). Alzheimer's Disease Neuroimaging Initiative (ADNI).

Supplementary Table 25. CSF t-tau levels across N/S (HCV/Ng) profiles in WRAP cohort

Group reported	Compared to	β	SE	T-value	P-value	Padj-value
N-S+	N-S-	5.285	1.155	11.559	<0.0001	<0.0001
N+S-	N-S-	0.983	1.155	-0.121	0.702	0.910
N+S+	N-S-	4.825	1.156	10.885	<0.0001	<0.0001
N+S-	N-S+	0.186	1.148	-12.176	<0.0001	<0.0001
N+S+	N-S+	0.913	1.141	-0.690	0.225	0.901
N+S+	N+S-	4.909	1.143	11.918	<0.0001	<0.0001
**Covariates**						
Age		1.026	1.007	3.55	<0.0001	-
Sex		1.171	1.11	1.519	0.13	-
Cognitive status		1.661	1.233	2.422	0.016	-
Aβ burden		1.051	1.042	1.206	0.229	-

Mean difference values of log-transformed and z-scored levels of t-tau comparing S and N profiles: N-S- (n = 55), N-S+ (n = 80), N+S- (n = 68), N+S+ (n = 82). Ng and HCV levels above median of CU A- individuals were used to classify individuals with abnormal synaptic dysfunction (S+) and abnormal neurodegeneration (N+). Mean differences were estimated with linear regression models adjusted for age, sex, cognitive status, amyloid burden (CSF $A\beta_{1-42}$), and cohort. Groups were added as dummy variables to assess differences in t-tau levels between groups, and pairwise comparisons were corrected for multiple testing using Tukey's method (Padj-value). A+ individuals were classified within each cohort according to previously published reference values (see Methods). Amyloid-β (A). Cognitively unimpaired (CU). Hippocampal volume (HCV). Neurogranin (Ng). Standard error (SE). CSF (cerebrospinal fluid). Total-tau (T-tau). Wisconsin Registry for Alzheimer's Prevention (WRAP).

Supplementary Table 26. CSF t-tau levels across N/S (HCV/SNAP25) profiles in WRAP cohort

Group reported	Compared to	β	SE	T-value	P-value	Padj-value
N-S+	N-S-	5.982	1.216	9.163	<0.0001	<0.0001
N+S-	N-S-	1.119	1.228	0.547	0.702	0.947
N+S+	N-S-	5.635	1.219	8.716	<0.0001	<0.0001
N+S-	N-S+	0.187	1.218	-8.509	<0.0001	<0.0001
N+S+	N-S+	0.942	1.198	-0.331	0.225	0.987
N+S+	N+S-	5.035	1.212	8.412	<0.0001	<0.0001
**Covariates**						
Age		1.027	1.01	2.718	0.007	-
Sex		0.851	1.158	-1.101	0.272	-
Cognitive status		1.89	1.374	2.004	0.047	-
Aβ burden		0.959	1.063	-0.69	0.491	-

Mean difference values of log-transformed and z-scored levels of t-tau comparing S and N profiles: N-S- (n = 34), N-S+ (n = 42), N+S- (n = 31), N+S+ (n = 49). SNAP25 and HCV levels above median of CU A- individuals were used to classify individuals with abnormal synaptic dysfunction (S+) and abnormal neurodegeneration (N+). Mean differences were estimated with linear regression models adjusted for age, sex, cognitive status, amyloid burden (CSF $A\beta_{1-42}$), and cohort. Groups were added as dummy variables to assess differences in t-tau levels between groups, and pairwise comparisons were corrected for multiple testing using Tukey's method (Padj-value). A+ individuals were classified within each cohort according to previously published reference values (see Methods). Amyloid-β (A). Cognitively unimpaired (CU). Hippocampal volume (HCV). Standard error (SE). CSF (cerebrospinal fluid). Total-tau (T-tau). Synaptosomal-associated protein 25 (SNAP25). Wisconsin Registry for Alzheimer's Prevention (WRAP).
