# Supplementary material for: CSF total tau as a proxy of synaptic degeneration
Source: Nat Commun. 2025 Aug 29;16:8076. doi: 10.1038/s41467-025-63545-5 (PMC12397218; doi:10.1038/s41467-025-63545-5)
Supplement: Supplementary file 2 — Reporting Summary [file 41467_2025_63545_MOESM2_ESM.pdf]

nature portfolio

Corresponding author(s): Tharick A. Pascoal

Last updated by author(s): Aug 14, 2025

Reporting Summary

Nature Portfolio wishes to improve the reproducibility of the work that we publish. This form provides structure for consistency and transparency in reporting. For further information on Nature Portfolio policies, see our Editorial Policies and the Editorial Policy Checklist.

Statistics

For all statistical analyses, confirm that the following items are present in the figure legend, table legend, main text, or Methods section.

n/a	Confirmed	
☐	☒	The exact sample size (*n*) for each experimental group/condition, given as a discrete number and unit of measurement
☐	☒	A statement on whether measurements were taken from distinct samples or whether the same sample was measured repeatedly
☐	☒	The statistical test(s) used AND whether they are one- or two-sided<br>*Only common tests should be described solely by name; describe more complex techniques in the Methods section.*
☐	☒	A description of all covariates tested
☐	☒	A description of any assumptions or corrections, such as tests of normality and adjustment for multiple comparisons
☐	☒	A full description of the statistical parameters including central tendency (e.g. means) or other basic estimates (e.g. regression coefficient) AND variation (e.g. standard deviation) or associated estimates of uncertainty (e.g. confidence intervals)
☐	☒	For null hypothesis testing, the test statistic (e.g. *F*, *t*, *r*) with confidence intervals, effect sizes, degrees of freedom and *P* value noted<br>*Give P values as exact values whenever suitable.*
☒	☐	For Bayesian analysis, information on the choice of priors and Markov chain Monte Carlo settings
☐	☒	For hierarchical and complex designs, identification of the appropriate level for tests and full reporting of outcomes
☐	☒	Estimates of effect sizes (e.g. Cohen's *d*, Pearson's *r*), indicating how they were calculated

Our web collection on statistics for biologists contains articles on many of the points above.

Software and code

Policy information about availability of computer code

Data collection	No specific code was used in data collection.
Data analysis	Demographic and unadjusted correlation statistical analyses were conducted using GraphPad Prism v.9, while the other statistical tests were performed using R-Studio Statistical software package 4.2.2.

For manuscripts utilizing custom algorithms or software that are central to the research but not yet described in published literature, software must be made available to editors and reviewers. We strongly encourage code deposition in a community repository (e.g. GitHub). See the Nature Portfolio guidelines for submitting code & software for further information.

Data

Policy information about availability of data

All manuscripts must include a data availability statement. This statement should provide the following information, where applicable:

- Accession codes, unique identifiers, or web links for publicly available datasets
- A description of any restrictions on data availability
- For clinical datasets or third party data, please ensure that the statement adheres to our policy

Inquiries regarding raw and analyzed data as well as materials can be directed to the corresponding author (T.A.P.). The investigators and affiliated institutions will promptly assess whether there are any intellectual property or confidentiality obligations associated with the request. Data from the ADNI cohort can be accessed from https://ida.loni.usc.edu. De-identified data from the WRAP cohort will be made accessible to qualified academic researchers upon request, specifically for the purpose of replicating the methods and findings outlined in this paper. More information from the WRAP cohort can be accessed from https://wrap.wisc.edu/. Any

releasable data and materials will be provided under a material transfer agreement. Please note that certain information is not publicly accessible to safeguard the privacy of the research participants. Source data is provided as a source data file. Source data are provided with this paper.
Results from this study were generated using R code, publicly accessible on https://github.com/PascoalLab/soares2025-ttau. All codes used open-source R packages.

Research involving human participants, their data, or biological material

Policy information about studies with human participants or human data. See also policy information about sex, gender (identity/presentation), and sexual orientation and race, ethnicity and racism.

Reporting on sex and gender	The findings presented here were observed in both men and women. Sex, not gender, was considered in the study design and was determined based on self-report. Sex was included as a covariate in all statistical models. No disaggregated sex or gender data are available for individual participants. No gender-based analyses were performed. We also performed association analysis stratified by sex.
Reporting on race, ethnicity, or other socially relevant groupings	The White population in the study corresponds to 92% in the cognitively unimpaired group and 94% in the cognitively impaired group. Additionally, the Non-hispanic population in the study corresponds to 96% in the cognitively unimpaired group and 97% in the cognitively impaired group.
Population characteristics	Table 1, and Supplemental Tables 1,2. We studied 1,692 individuals, including 760 cognitively unimpaired (CU) and 932 cognitively impaired (CI) individuals . Briefly, the mean age was 70.9 years old (s.d 7.8), 50.9% females, from two research-based cohorts (1,407 from ADNI and 285 from WRAP).
Recruitment	The Alzheimer's Disease Neuroimaging Initiative (ADNI) is a multicenter, longitudinal study that began in 2004 to identify and track biomarkers of Alzheimer's disease. Participants aged 55 to 90 were recruited across the cognitive spectrum—including cognitively normal individuals, those with mild cognitive impairment (MCI), and those with mild Alzheimer's disease—through memory clinics, community outreach, and advertisements. Eligibility criteria included good general health, stable medication use, adequate sensory abilities for testing, and the availability of a study partner. Specific cognitive and clinical criteria were used to classify participants into diagnostic groups, and efforts were made to enhance diversity by recruiting individuals from underrepresented populations. The Wisconsin Registry for Alzheimer's Prevention (WRAP) is a longitudinal cohort study that began in 2001, recruiting cognitively healthy adults aged 40–65, primarily enriched for individuals with a parental history of Alzheimer's disease. Participants were recruited through memory clinics, community outreach, advertisements, and word-of-mouth. Eligibility required good general health, English fluency, and no dementia diagnosis at baseline.
Ethics oversight	The ADNI database provides clinical assessments, neuroimaging, and biomarker measurements, and regional ethical committees of all institutions approved the study. All individuals provided informed consent. The WRAP study was approved by the University of Wisconsin Institutional Review Board and all participants provided signed informed consent before participation.

Note that full information on the approval of the study protocol must also be provided in the manuscript.

Field-specific reporting

Please select the one below that is the best fit for your research. If you are not sure, read the appropriate sections before making your selection.

[x] Life sciences [ ] Behavioural & social sciences [ ] Ecological, evolutionary & environmental sciences

For a reference copy of the document with all sections, see nature.com/documents/nr-reporting-summary-flat.pdf

Life sciences study design

All studies must disclose on these points even when the disclosure is negative.

Sample size	We selected the two cohorts with the highest concentrations of synaptic markers in the cerebrospinal fluid (CSF), ensuring relevance and robustness for our analysis. These cohorts were chosen based on data availability and biological significance, aligning with our study's objectives. For the primary analysis, which we test the association of CSF t-tau with synaptic and neurodegeneration biomarkers, we estimated that a sample size of 57 would provide 90% statistical power at a 5% alpha level assuming a medium effect size (f2 = 0.15). However, rather than limiting the dataset to a predefined sample size, we included two cohorts for validation and all available data from both cohorts that met our inclusion criteria. This approach maximizes statistical power and generalizability while minimizing selection bias. In summary, the number of datasets was determined by both biological relevance and data availability and is sufficient to support the planned analyses with high statistical confidence.<br>Accordingly, we studied 1,692 individuals, including cognitively unimpaired (CU), MCI and individuals with dementia due to AD, obtained from two cohorts: the Alzheimer's disease Neuroimaging Initiative (ADNI; N = 1,407) database (adni.loni.usc.edu) a multi-center research effort initiated in 2004, and the Wisconsin Registry for Alzheimer's Prevention study (WRAP; N = 285), based in Wisconsin, USA (https://wrap.wisc.edu).
Data exclusions	Participants were included if they had > 50 years old and had a clinical diagnosis or CDR, cerebrospinal fluid (CSF) t-tau, CSF Amyloid-beta1-42, and at least one of the following biomarkers: HCV, CSF NfL, CSF Ng, CSF SNAP25, tau-PET. CSF measures from ADNI with notes indicating potential analytical problems (e.g. above limit of quantification) were removed from analysis.
Replication	Our results were replicated in two independent cohorts.

Randomization	As these are observational cohort studies, participants were not randomized into experimental groups. Therefore, randomization does not apply. However, statistical models accounted for potential confounding factors, including age and sex.
Blinding	All biomarker analyses (including PET and fluid measures) were conducted without knowledge of participants' clinical diagnoses to ensure blinding.

Reporting for specific materials, systems and methods

We require information from authors about some types of materials, experimental systems and methods used in many studies. Here, indicate whether each material, system or method listed is relevant to your study. If you are not sure if a list item applies to your research, read the appropriate section before selecting a response.

Materials & experimental systems

n/a	Involved in the study
☒	☐ Antibodies
☒	☐ Eukaryotic cell lines
☒	☐ Palaeontology and archaeology
☒	☐ Animals and other organisms
☒	☐ Clinical data
☒	☐ Dual use research of concern
☒	☐ Plants

Methods

n/a	Involved in the study
☒	☐ ChIP-seq
☒	☐ Flow cytometry
☒	☐ MRI-based neuroimaging

Plants

Seed stocks	*Report on the source of all seed stocks or other plant material used. If applicable, state the seed stock centre and catalogue number. If plant specimens were collected from the field, describe the collection location, date and sampling procedures.*
Novel plant genotypes	*Describe the methods by which all novel plant genotypes were produced. This includes those generated by transgenic approaches, gene editing, chemical/radiation-based mutagenesis and hybridization. For transgenic lines, describe the transformation method, the number of independent lines analyzed and the generation upon which experiments were performed. For gene-edited lines, describe the editor used, the endogenous sequence targeted for editing, the targeting guide RNA sequence (if applicable) and how the editor was applied.*
Authentication	*Describe any authentication procedures for each seed stock used or novel genotype generated. Describe any experiments used to assess the effect of a mutation and, where applicable, how potential secondary effects (e.g. second site T-DNA insertions, mosiacism, off-target gene editing) were examined.*
